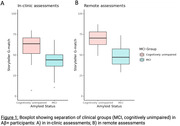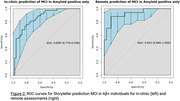# Predicting Mild Cognitive Impairment in Amyloid Beta Biomarker Positive Individuals Using Automated Speech‐Based Testing Procedures

**DOI:** 10.1002/alz.093319

**Published:** 2025-01-09

**Authors:** Caroline Skirrow, Udeepa Meepegama, Michael T. Ropacki, Jack Weston, Emil Fristed

**Affiliations:** ^1^ Novoic, London United Kingdom; ^2^ Strategic Global Research & Development, Temecula, CA USA

## Abstract

**Background:**

New disease modifying treatments for Alzheimer’s Disease (AD) require confirmation of amyloid biomarkers and evidence of clinical and/or cognitive AD symptoms. Blood tests for AD biomarkers are poised to enter routine care. However, at‐risk individuals with positive biomarkers vary in their level of cognitive impairment, raising the need for sensitive and scalable cognitive assessments. Fully automated speech‐based testing to screen for Mild Cognitive Impairment (MCI) has shown promise to bridge this gap, but its efficacy specifically within amyloid beta positive (Aβ+) individuals is yet to be fully established.

**Method:**

Data from Aβ+ participants in the AMYPRED‐UK (NCT04828122) and AMYPRED‐US studies (NCT04928976) with Clinical Dementia Score (CDR‐G) between 0‐0.5 were analyzed. Speech recordings from Storyteller, a test battery leveraging two immediate and one delayed recall conditions from Automated Story Recall Task (ASRT), were collected from 84 Aβ+ individuals (n=38 MCI; n=46 unimpaired) who completed in‐clinic visits. A subsample of 46 Aβ+ participants (n=17 MCI; n=29 unimpaired) also completed Storyteller in a separate optional remote assessment session. Spoken responses were automatically transcribed and analysed to obtain a measure of proportional recall, “G‐match”, averaged over the three recall trials separately for in‐clinic and remote sessions. Analyses included T‐test for quantifying group differences, and prediction of MCI using the Area Under the Receiver Operating Characteristic curve (AUC). Correlations with the Wechsler Logical Memory Delayed Recall (LMDR) test and the Preclinical Alzheimer’s Cognitive Composite with semantic processing (PACC5) were also evaluated.

**Result:**

G‐match scores differed between MCI and cognitively unimpaired participants with large effect sizes for both in‐clinic (Cohen’s d=1.36) and remote assessments (d=2.07), Figure 1. Predictive ability for MCI was shown with AUC of 0.83 and 0.92 for in‐clinic and remote assessments respectively (Figure 2). Moderate‐to‐high correlations were observed between G‐match, LMDR, and PACC5 outcomes (r=0.73‐0.84).

**Conclusion:**

Speech‐based screening shows good sensitivity for detecting MCI in at‐risk populations. This approach could support at‐scale screening for suitability for new disease modifying treatments and for new treatment and prevention trials, with assessments incorporated into routine clinical visits or completed remotely at home.